# Adsorption of Urinary Proteins on the Conventionally Used Urine Collection Tubes: Possible Effects on Urinary Proteome Analysis and Prevention of the Adsorption by Polymer Coating

**DOI:** 10.1155/2011/502845

**Published:** 2011-09-12

**Authors:** Iwao Kiyokawa, Kazuyuki Sogawa, Keiko Ise, Fumie Iida, Mamoru Satoh, Toshihide Miura, Ryo Kojima, Katsuhiro Katayama, Fumio Nomura

**Affiliations:** ^1^Clinical Proteomics Research Center, Chiba University Hospital, Chiba, Japan; ^2^R&D Department, Nittobo Medical Co., Ltd., Fukushima, Koriyama City 963-8061, Japan; ^3^Department of Clinical Laboratory, Chiba University Hospital, Chiba, Japan; ^4^Department of Molecular Diagnosis (F8), Graduate School of Medicine, Chiba University, Chiba 260-8670, Japan

## Abstract

One possible factor determining recovery of trace amount of protein biomarker candidates during proteome analyses could be adsorption on urine tubes. This issue, however, has not been well addressed so far. Recently, a new technical device of surface coating by poly(2-methacryloyloxyethyl phosphorylcholine (MPC)-*co*-n-butyl methacrylate (BMA)) (poly(MPC-*co*-BMA)) has been developed mainly to prevent the adsorption of plasma proteins. We assessed whether conventionally used urine tubes adsorb trace amount of urinary proteins and, if any, whether the surface coating by poly(MPC-*co*-BMA) can minimize the adsorption. Proteinuric urine samples were kept in poly(MPC-*co*-BMA)-coated and noncoated urine tubes for 15 min and possibly adsorbed proteins and/or peptides onto urine tubes were analyzed by SDS-PAGE, 2-DE, and the MALDI-TOF MS. It was found that a number of proteins and/or peptides adsorb on the conventionally used urine tubes and that surface coating by poly(MPC-*co*-BMA) can minimize the adsorption without any significant effects on routine urinalysis test results. Although it remains to be clarified to what extent the protein adsorption can modify the results of urinary proteome analyses, one has to consider this possible adsorption of urinary proteins when searching for trace amounts of protein biomarkers in urine.

## 1. Introduction

Urine has now become one of the most attractive biological fluids in clinical proteomics [1, 2]. A number of urinary proteomic studies have been conducted and have revealed urinary biomarker candidates for renal systemic diseases and malignancies of urinary tract [3–5]. Proteomic analysis of urines can be applied to biomarker search in nonrenal diseases as well [6–8].

Although urinary proteome analyses have been conducted by various gel-based and gel-free techniques [9], comprehensive urinary proteome analysis is not an easy task because the urine has very diluted protein concentration with high levels of salts. Sample preparation, processing, and storage for urinary proteomics have been reviewed [10–13]. More recently, an optimized quantitative proteomic strategy for urine biomarker discovery was described [14]. In any event, maximal protein recovery from urine is essential for detecting trace quantities of proteins present in urine for potential biomarker discovery. For this purpose, protein loss during sample preparation should be avoided. One possible factor responsible for loss of trace amounts of urinary proteins could be adsorption to sample tubes, but this issue has not been well addressed so far to our knowledge.

Many attempts have been made to prevent the adsorption of plasma proteins and to improve blood compatibility by surface modification [15, 16]. Ishihara and coworkers reported on rapid development of hydrophilicity and protein adsorption resistance by polymer surfaces bearing poly(2-methacryloyloxyethyl phosphorylcholine (MPC)*-co-*n-butyl methacrylate) (poly(MPC*-co-*BMA)) [17, 18].

We took advantage of this coating method in the present study and assessed whether conventionally used tubes adsorb trace amount of urinary proteins and, if any, whether the surface coating by poly(MPC*-co-*BMA) can minimize the adsorption.

## 2. Materials and Methods

### 2.1. Urine Collection Tubes and Coating Method

Poly(MPC*-co-*BMA) was obtained from AI BIO-CHIPS CO., LTD (Tokyo, Japan). A total of 6 different types of conventional urine collection tubes were used in this study. Tubes made from polystyrene (PS) (Cat# 10200), polypropylene(PP) Cat# 72200, polyethylene terephthalate(PET) (Cat# 23540), and styrene-butadiene copolymers/methyl methacrylate-styrene (SBC/MS) (Cat# 17300) were purchased from TOYO KAGAKU KIZAI Co., LTD., Japan. Tubes made from acrylonitrile-styrene (AS) copolymers (Cat# 479511373) were from Nittobo Medical Co., LTD., Japan and those made from styrene-butadiene copolymers (SBC) (Cat# 3324A000A-10) were from ASIAKIZAI Co., LTD., Japan. The conventional tubes made by AS were coated by poly(MPC*-co-*BMA) as described by Futamura et al. [18]. 

### 2.2. Samples

Urine samples obtained from outpatients in Chiba University Hospital were used. An aliquot of the samples was taken for routine urinalysis, and the rest of the samples were centrifuged (700 ×g, 5 min at room temperature), and the supernatant was subjected to assess protein adsorption on test tubes as described below. All these procedures were carried out within 2 hours after collection of the samples.

### 2.3. Urine Sample Preparation for Electrophoresis (SDS-PAGE and 2-DE) and MALDI-TOF MS

One mL of two different levels of pooled proteinuric urines (equivalent to 15 mg/dL and 50 mg/dL, resp.) obtained from 10 patients with renal disease were put into poly(MPC*-co-*BMA)-coated and noncoated urine correction tubes and were kept at room temperature for 15 min. After aspiration of the urines, the tubes were washed with 200 **μ**L of PBS three times. After the third wash and PBS being aspirated, 100 **μ**L of PAGE sample buffer (electrophoresis) (50 mM Tris-HCl, pH 6.8 containing 50 mM dithiothreitol, 0.5% SDS, and 10% glycerol) or 1% TCA aqueous solution (MALDI-TOF MS analysis) was added and the tubes were vortexed for 30 sec to dissolve possibly adsorbed proteins.

### 2.4. Gel-Based Analysis

The solution which contained proteins possibly adsorbed on the urine tubes was then analyzed using SDS-PAGE (Perfect NT Gel W, 10–20% acrylamide, 20 wells; DRC Co., Ltd., Tokyo, Japan) according to the manufacturer's protocol. The gel was stained with CBB (PhastGel Blue R; GE Healthcare, Little Chalfont, UK). The proteins separated by SDS-PAGE were identified by in-gel tryptic digestion of the proteins followed by MS. In-gel tryptic digestion was performed as described previously [19]. Molar quantities of recovered peptide fragments were estimated from the staining intensity of the SDS-PAGE bands that were digested in-gel with trypsin. Digested peptides roughly equivalent up to 1 pmol of protein were injected into a trap column: 0.3 × 5 mm L-trap column (Chemicals Evaluation and Research Institute, Saitama, Japan), and an analytical column: 0.1 × 50 mm Monolith column (AMR, Tokyo, Japan), which was attached to a HPLC system (Nanospace SI-2; Shiseido Fine Chemicals, Tokyo, Japan). The flow rate of a mobile phase was 1 **μ**L/min. The solvent composition of the mobile phase was programmed to change in 35 min cycles with varying mixing ratios of solvent A (2% v/v CH_3_CN and 0.1% v/v HCOOH) to solvent B (90% v/v CH_3_CN and 0.1% v/v HCOOH): 5–50% B 20 min, 50–95% B 1 min, 95% B 3 min, 95–5% B 1 min, 5% B 10 min. Purified peptides were introduced from HPLC to an LTQ-XL (Thermo Scientific, Calif, USA), an ion trap mass spectrometer (ITMS), via an attached Pico Tip (New Objective, Mass, USA). The MS and MS/MS peptide spectra were measured in a data-dependent manner according to the manufacturer's operating specifications. The Mascot search engine (Matrix science, London, UK) was used to identify proteins from the mass and tandem mass spectra of peptides. Peptide mass data were matched by searching the Human International Protein Index database (IPI, July 2008, 72079 entries, European Bioinformatics Institute) using the MASCOT engine. The minimum criterion of the probability-based MASCOT/MOWSE score was set with 5% as the significant threshold level.

For 2-DE analysis, we used the method described by Oh-Ishi et al. [20] and Kawashima et al. [21]. Briefly, one mL aliquots of urine samples kept at room temperature for 15 min in poly(MPC*-co-*BMA)-coated and noncoated urine correction tube were concentrated up to 20-fold by BJP Concentrator (ProChem, MA, USA) to 50 **μ**L and lyophilized. The lyophilizate was resuspended by 200 **μ**L of Immobiline reagent (7 M urea, 2 M thiourea, 4% CHAPS, 2% DTT, 2% Pharmalyte, broad range pH 3–10). Finally, 50 **μ**L of the 5-fold urine sample was applied to the IEF agarose gel. The agarose gel was then transferred to the Perfect NT Gels W (10–20% gradient of polyacrylamide gel; DRC. Co. Ltd, Tokyo, Japan) and the second electrophoresis was performed. Protein spots on 2-DE gels were stained with CBB. The protein spots were detected, quantified, and matched with the 2-DE gel view analysis software, Progenesis SameSpots (Nonlinear Dynamics Ltd., UK). The protein spots were excised from the gel and identified, as we previously described [19].

### 2.5. MS-Based Analysis

One mL aliquots of urine samples (containing 50 mg/mL protein) were kept at room temperature for 15 min in poly(MPC*-co-*BMA)-coated and noncoated urine collection tube. Proteins possibly adsorbed on the tubes were collected as described above for the gel-based method and were analyzed by the MALDI-TOF MS. To obtain quantitative data of the possibly adsorbed proteins, we used stable isotope-labeled 5.9 kDa fibrinogen alpha C chain fragment (FIC 5.9) as an internal standard as described by Sogawa et al. [22]. We obtained the stable isotope-labeled synthetic FIC 5.9 from the AnyGen Co., Ltd. (Kwangju, Korea). The amino acid sequence of the peptide was SSSYSKQFTSSTSYNRG DSTFESKSYKMADEAGSEADHEGTHSTKRGHAKSRPV. (The underlined amino acids were synthesized with ^13^C, ^15^N uniformly labeled FMOC amino acids.). In urine analysis, ten microliters of SID (stable isotope-labeled) -FIC 5.9 solution (0.5 pmol/**μ**L SID-FIC 5.9, MB-WCX binding solution) and 5 **μ**L of urine sample were transferred to a 200 **μ**L PCR tube (Thermo Fisher Scientific K.K., Kanagawa, Japan). In analysis of urine samples kept in tube, ten microliters of SID-FIC 5.9 solution (0.025 pmol/**μ**L SID-FIC 5.9, MB-WCX binding solution) and 5 **μ**L of extracted samples (urine samples kept in tube) were transferred to a 200 **μ**L PCR tube. A 10 **μ**L homogenous magnetic particle solution was added, mixed with the other solutions, and allowed to sit for 5 min. The PCR tubes were placed in a magnetic bead separator (MBS; Bruker Daltonics GmbH) for 30 s for magnetic fixation of the MB-WCX particles. The supernatant was aspirated, and the tubes were removed from the MBS device. We added 100 **μ**L of the washing solution and carefully mixed it with the magnetic beads. We then replaced the tube into the MBS device and moved it back and forth between adjacent wells on each side of the device's magnetic bar. After fixation of the magnetic beads for 30 s, the supernatant was aspirated. We repeated this washing procedure three times. After the final wash, we eluted the bound molecules by incubating them for 1 min with 5 **μ**L MB-WCX elution solution and then used the MBS device to collect the eluate. For the final step, we added 5 **μ**L of the MB-WCX stabilization solution to the eluate. We then mixed 1 **μ**L of the eluate with 5 **μ**L of a matrix solution (0.3 g/L a-cyano-4-hydroxycinnamic acid in ethanol:acetone, 2 : 1). We spotted 1 **μ**L of the mixture onto an AnchorChip target plate (Bruker Daltonics GmbH) and allowed it to dry. Protein Calibration standard (Protein Calibration standard 1, Bruker Daltonics GmbH) was dissolved in 125 1 **μ**L. We applied 0.5 1 **μ**L of the solution to target spots in proximity to the urine samples for external calibration.

We placed the AnchorChip target plate into the AutoFlex II TOF/TOF mass spectrometer (Bruker Daltonics GmbH) and into the UltraFlex III TOF/TOF mass spectrometer (Bruker Daltonics GmbH), which is controlled by Flexcontrol software 3.0 (Bruker Daltonics GmbH). The instrument was externally calibrated by standard procedures. The automated acquisition method included in the instrument software generated all acquisitions. The automated acquisition laser power was set between 25% and 35%. Spectra were acquired in a positive linear mode in the mass range of 600 to 10,000 Da.

We used FlexAnalysis software 3.0 to perform baseline correction and smoothing. The concentration of the proteins adsorbed to urine collection tube was estimated from the ratio of the peak intensity of adsorbed proteins to the peak intensity of SID-FIC 5.9. For identification of peptides as we previously described [23], the AnchorChip target plate was also placed in an UltrafleXtreme TOF/TOF mass spectrometer (Bruker Daltonics) and the MALDI-TOF/TOF MS/MS spectrum was recorded in LIFT mode. Five hundred laser shots from a total of 3000 laser shots were summed. The MALDI-TOF/TOF MS/MS spectrum was subjected to a database search using the Mascot (Matrix Science, London, UK) database search engine. The search parameters were as follows: no enzyme specificity, 25 ppm mass tolerance for the parent mass, and 0.2 Da for fragment masses. No fixed or variable modifications were selected. The NCBInr database was used for the search. 

### 2.6. Urinalysis Testing

#### 2.6.1. Quantitative Study

One hundred urine samples requested for urinalysis on routine basis at the Division of Laboratory Medicine and Clinical Genetics, Chiba University Hospital were used. The urine samples were aliquoted (10 mL) to poly(MPC*-co-*BMA)-coated and noncoated collection tubes and were kept at room temperature for 15 min before use. Nine different quantitative urinalysis such as protein, glucose, creatinine, microalbumin, beta-2-microglobulin, amylase, *N*-acetyl-*β*-D-glucosaminidase, urea nitrogen, uric acid, and six kinds of electrolytes were conducted using BioMajesty JCA-BM6010 (JEOL Ltd., Tokyo, Japan).

#### 2.6.2. Dipstick Urinalysis

The Uriflet S-9UB (Arkray Inc., Tokyo, Japan) and AUTION MAX AX-4030 (Arkray Inc., Tokyo, Japan) analyzers were used. Ten different parameters are assessed: specific gravity (SG, measured via a built-in refractometer), erythrocytes, leukocytes, nitrite, pH, protein, glucose, ketones, bilirubin, and urobilinogen.

#### 2.6.3. Urinary Sediments

The AUTION IQ IQ-5210 analyzer (Arkray Inc., Tokyo, Japan) was used to determine urinary sediments. This equipment includes digital imaging and Auto-Particle Recognition (APR) (Chatsworth, CA, USA) software to classify urine particles and quantitatively report results. In this study, 4 categories red blood cells (RBC), white blood cells (WBC), squamous epithelial cells (SEC), and casts were classified by the APR software.

### 2.7. Statistical Analysis

The numerical data are presented as the mean ± standard deviation (SD). We evaluated the statistical significance using IBM SPSS Statistics 18 software (SPSS Inc., IL, USA). A *P* < 0.05 was considered significant using the Mann-Whitney *U *test.

## 3. Results

### 3.1. Detection of Urinary Proteins Adsorbed on Urine Tubes by SDS-PAGE

**Figure 1:**
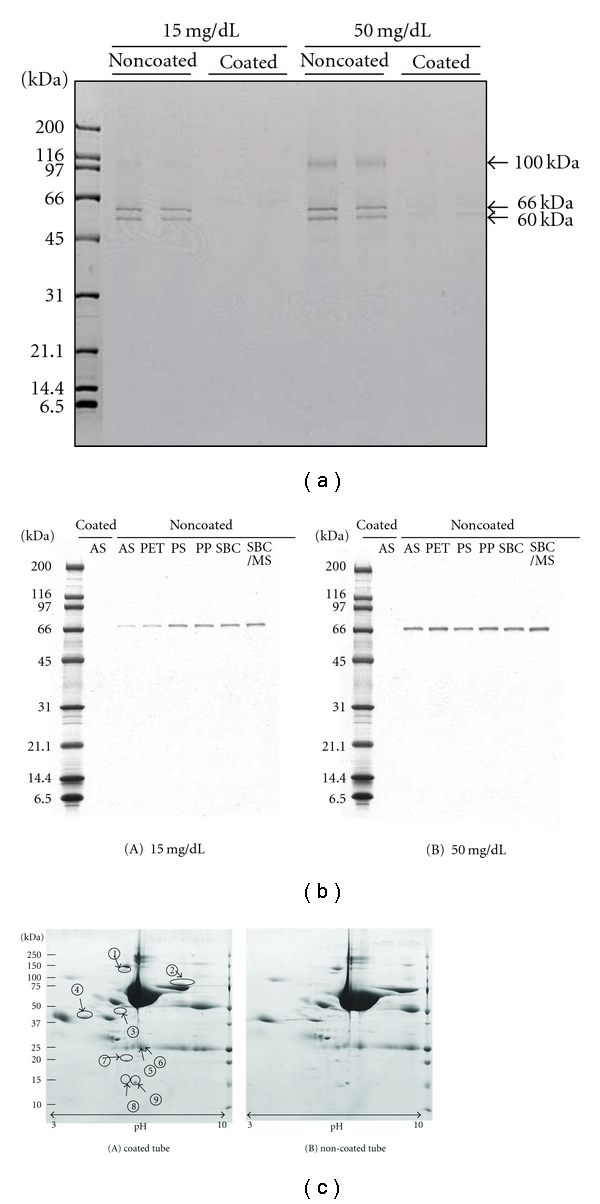
(a) SDS-PAGE of adsorbed proteins to poly(MPC*-co-*BMA)-coated and noncoated urine tubes in two different grades of proteinuric samples. A few distinct protein bands are noted in samples obtained from noncoated tubes. Similar results were obtained in 9 different experiments. (b) SDS-PAGE of adsorbed proteins to 6 different types of poly(MPC*-co-*BMA) noncoated conventionally used urine collection tubes. AS: poly(acrylonitrile-styrene), PET: polyethylene terephthalate, PS: polystyrene, PP: polypropylene, SBC: styrene-butadiene, and SBC/MS: styrene-butadiene copolymers/methyl methacrylate-styrene. (c) 2-DE of urinary proteins obtained from samples kept at poly(MPC*-co-*BMA)-coated and noncoated urine tubes. Nine protein spots the intensities of which were significantly greater (*P* < 0.05) in samples kept at poly(MPC*-co-*BMA)-coated tubes compared with those kept at noncoated tubes were selected based on the results obtained in seven different experiments. The 2-DE gels are shown for pH 3–10.

Proteins adsorbed on the poly(MPC*-co-*BMA)-coated and noncoated tubes were analyzed by SDS-PAGE. As shown in Figure 1(a), a few distinct protein bands (60 kDa, 66 kDa and 100 kDa) were noted in samples obtained from noncoated AS tubes. No clear bands were visible in samples obtained from poly(MPC*-co-*BMA)-coated AS tubes under these experimental conditions. LC-MS analysis of trypsin digests of these bands identified 11 proteins as listed in Table 1. Protein adsorption on the tubes was observed in 6 different types of conventionally used urine collection tubes as shown in Figure 1(b).

### 3.2. 2-DE Analysis of Urine Samples Kept in Poly(MPC-co-BMA)-Coated and Noncoated Tubes

Urine specimens kept in poly(MPC*-co-*BMA)-coated and noncoated tubes were subjected to the agarose 2-DE as described in the Methods section. The representative patterns were presented in Figure 1(c). Nine protein spots the intensities of which were significantly greater (*P* < 0.05) in samples kept at poly(MPC*-co-*BMA)-coated tubes compared with those kept at noncoated tubes were selected based on the results obtained in seven different experiments.

These differences were most likely as the results of more protein adsorption on noncoated tubes. These spots were excised and subjected to in-gel trypsin digestion followed by LC-MS. A total of 15 proteins were identified as listed in Table 2.

### 3.3. MALDI-TOF MS Analysis of Urinary Proteins Adsorbed on Poly(MPC-co-BMA)-Coated and Noncoated Tubes

**Figure 2:**
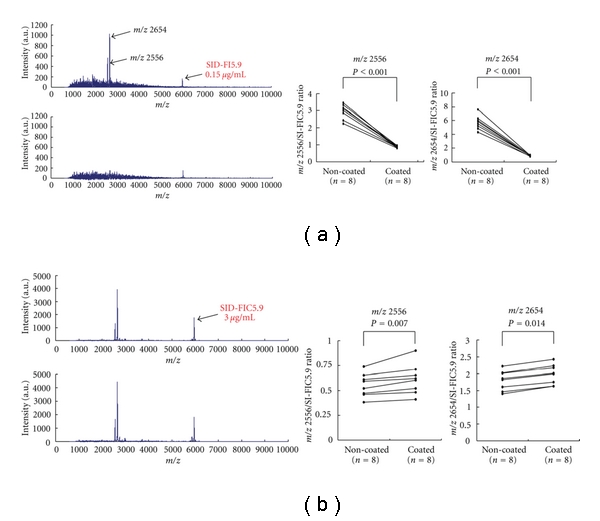
Representative spectra of the MALDI-TOF MS of urinary proteins and/or peptides adsorbed on urinary tubes with and without poly(MPC*-co-*BMA) coating (a) and those of urine samples kept in the coated and noncoated tubes (b). (a) Left: it was notable that 2556 m/z and 2645 m/z peaks were observed in proteinuric samples only when obtained from the poly(MPC-*co*-BMA) non-coated tubes (the upper panel). Similar results were obtained in 7 different experiments. Right: the two peaks detectable in noncoated tubes were significantly attenuated in poly(MPC*-co-*BMA)-coated tubes (*P* < 0.001). (b) When urinary samples were kept in the coated and uncoated urine tubes, relative peak intensities of the 2556 m/z and 2645 m/z peaks were attenuated in poly(MPC*-co-*BMA)-coated tubes (the upper panel), which was confirmed by the quantitative study using the internal standard (2556 m/z; *P* < 0.007, 2653 m/z; *P* < 0.014).

Proteins and/or peptides adsorbed on the conventional urine tubes were also detectable by the MALDI-TOF MS.  Figure 2(a) shows a representative spectrum of the adsorbed proteins and peptides. The intensities of the two peaks (2556 m/z and 2654 m/z) were notably greater in samples obtained from poly(MPC*-co-*BMA) noncoated tubes. Similar results were obtained in 7 different experiments; the expression levels of the two peaks expressed as the ratio to the internal standard, SID-FIC 5.9, were significantly greater (*P* < 0.001) in poly(MPC*-co-*BMA) noncoated tubes than in coated tubes. Using MALDI-TOF/TOF MS/MS technology, we successfully identified the two peaks (2556 m/z and 2654 m/z) as internal sequences of the fibrinogen alpha C chain fragment. The peptide sequences of the 2556 m/z and 2654 m/z were DEAGSEADHEGTHSTKRGHAKSRP and DEAGSEADHEGTHSTKRGHAKSRPV, respectively. The mean value of the ratio of m/z 2654 to SID-FIC5.9 was 5.70 (Figure 2(a) Right panel) in poly(MPC*-co-*BMA) noncoated urine collection tube. It is 0.85 *μ*g/mL when converting it into the protein concentration.

### 3.4. MALDI-TOF MS Analysis of Urine Samples Kept in Poly(MPC-co-BMA)-Coated and Noncoated Tubes

Figure 2(b) shows representative view of the spectrum of urine samples kept in urine tubes. The relative intensities of the two peaks (2556 m/z and 2654 m/z) were greater in the samples kept in poly(MPC*-co-*BMA)-coated tubes than those kept in noncoated tubes. Similar results were obtained in 7 other experiments; the differences quantified using the SID-FIC 5.9 were statistically significant (*P* < 0.007 for 2556 m/z and *P* < 0.014 for 2653 m/z).

### 3.5. Routine Urinalysis

The quantitative values of urinalysis parameters in samples kept in poly(MPC*-co-*BMA)-coated and noncoated collection tubes are presented in Table 3. They were all comparable between the two groups in linear regression equation, slope of linearity, correlation coefficients ranged from 0.997 to 1.014. The results of dipstick urinalysis and urinary sediment determinations were also comparable between the two groups.

## 4. Discussion

The issue of preanalytical factors affecting sample integrity is often overlooked and yet is critically important. Although preanalytical factors for serum or plasma proteome analysis have been extensively studied, the impact of adsorption of proteins and peptides on urine tubes on biomarker discovery using urinary proteomics is not well investigated.

The results of this study indicate that conventionally used urine collection tubes adsorb proteins and/or peptides and that the surface coating of the tubes by poly(MPC*-co-*BMA) can minimize the adsorption without any significant effects on routine chemical determinations.

In this study, urine samples were kept in poly(MPC*-co-*BMA)-coated and noncoated tubes for 15 min. This is because it is generally known that proteins adsorb onto a surface within a few minutes when the material contacts body fluids such as blood, plasma, and tears [24–26].

Protein adsorption is one of the most important phenomena in determination of the biocompatibility of materials [16, 18]. Several methods have been proposed to reduce protein adsorption on medical devices.

Polymers composed of MPC and hydrophobic alkylmethacrylate units have been extensively used in many medical devices as coating materials to improve the blood compatibility of these devices [15–18]. However, this coating requires a long wetting pretreatment time to achieve equilibrium hydration by the reorientation of the phosphorylcholine groups [16, 27]. In this study, urinary proteins were found to be adsorbed on poly(MPC*-co-*BMA) noncoated urine collection tubes made from six different types of materials. Recently, Futamura et al. [18] succeeded in rapid development of hydrophilicity and protein adsorption resistance poly(ethylene terephthalate) (PET) surfaces bearing poly(MPC*-co-*2-vinylnaphthalene(vN)) (PMvN). It should be considered, however, that coating effects on the plastic tubes appear to be dependent on the initial properties of the plastic tubes. We took advantage of this coating method and showed that protein adsorption can be reduced in urine samples as well. 

Most of the proteins listed in Table 1 are representative protein in urine and have theoretical isoelectric points between 4.7 and 7.0, suggesting that proteins with isoelectric point of this range are likely to be adsorbed on the conventional urine tubes employed in this study. Since the pH of the urine samples kept in poly(MPC*-co-*BMA)-coated and noncoated tube was similar, it is unlikely that the differences obtained in this study were due to pH difference. It has been reported that the factor responsible for protein adsorption to the plastic tube might depend on the relation of sample pH and protein pI [28].

Three proteins (alpha-1-antitrypsin, IGKV1-5 protein, prostaglandin-H2 D-isomerase) were detected in common for two different comparisons.

Ceruloplasmin, one of the proteins listed in Table 1, is a biomarker of uranium nephrotoxicity [29].

The use of the coated tubes did not have any impact on the urine analysis of routine parameters. Since there were no significant differences in the quantitative data of abundant urinary proteins including albumin and beta-2-microglobulin, the effects of adsorption on abundant proteins may be minimal. But, in searching for urinary protein biomarkers with low concentration, possible adsorption on conventional urine tubes should be considered. Since the material used in the conventional and noncoated tubes employed in the present study is widely used around the world, possible adsorption of trance amount of proteins to urine collection tubes should be considered in proteome analyses of urine samples.

## 5. Summary

Urine is one of the attractive biofluids in clinical proteomics. In chasing very low abundance urinary proteins and peptides, however, loss of biomarker candidates by adsorption on urine tubes should be considered. In this study, we found that protein adsorption on the conventionally used urine collection tubes is not negligible, and that the adsorption can be reduced by using a tube coated by hydrophilic polymers without any effects on routine urinalysis. 

I believe that these findings should be shared by those who are interested in urinary proteomic study.

## Figures and Tables

**Table 1:** Proteins adsorbed on poly(MPC-*co*-BMA)-uncoated urine tubes.

No.	ID	M.W.^a^	Score	Queries matched	pI^b^
1	Tamm-Horsfall urinary glycoprotein	69,761 Da	250	15	4.96
2	Albumin	69,321 Da	353	32	5.67
3	Semenogelin-1	52,131 Da	114	6	9.26
4	Alpha-1-antichymotrypsin	47,651 Da	281	11	5.32
5	Alpha-1-antitrypsin	46,737 Da	85	3	5.37
6	Apolipoprotein A1	27,891 Da	82	4	5.27
7	IGKV1-5 protein	25,765 Da	313	7	5.74–6.30
8	Prostaglandin-H2 D-isomerase	21,029 Da	98	5	8.37
9	Apolipoprotein C3	10,846 Da	93	3	4.72
10	Protein S100-A8	10,835 Da	86	3	6.51
11	SH3 domain-binding glutamic acid-rich-like protein 3	10,438 Da	80	2	4.82

^a,b^Theoretical Mr and pI, as resulted from Compute pI/Mw tool of Expasy (http://us.expasy.org/tools/pi_tool.html), are also indicated.

**Table 2:** Proteins which were reduced when kept in poly(MPC*-co-*BMA) noncoated urine tubes.

No.	ID	M.W.^a^	Score	Queries matched	pI^b^
1	Ceruloplasmin	122,128 Da	812	52	5.41
2	Lysosomal alpha-glucosidase	105,271 Da	152	8	5.41
3	Alpha-N-acetylglucosaminidase	82,115 Da	163	6	6.21
4	Serotransferrin	77,000 Da	1500	95	6.70
5	Alpha-1-antitrypsin	46,707 Da	253	12	5.37
6	Cell adhesion molecule 4	42,759 Da	134	6	5.59
7	Prostate-specific antigen	28,723 Da	80	2	7.26
8	IGK protein	26,218 Da	1416	4	5.74–6.30
9	IGL protein	24,777 Da	430	31	5.74–6.30
10	Alpha-1-acid glycoprotein 1	23,497 Da	239	7	5.00
11	Prostaglandin 2D synthase	22,932 Da	596	31	7.66
12	Prostaglandin-H2 D-isomerase	21,029 Da	861	42	7.66
13	Transthyretin	15,877 Da	856	37	5.35
14	Rheumatoid factor D5 light chain	12,758 Da	273	6	5.74–6.30
15	Rheumatoid factor D6 light chain	12,520 Da	273	6	5.74–6.30

^a,b^Theoretical Mr and pI, as resulted from Compute pI/Mw tool of Expasy (http://us.expasy.org/tools/pi_tool.html), are also indicated.

**Table 3:** Quantitative values for urinalysis parameters in samples kept in poly(MPC-*co*-BMA)-coated and noncoated urine collection tubes.

Parameters	Noncoated tube	Coated tube
pH	6.17 ± 0.58	6.17 ± 0.58
Protein (mg/dL)	57.8 ± 136.5	58.5 ± 138.2
Glucose (mg/dL)	182.4 ± 556.3	184.2 ± 561.0
Creatinine (mg/mL)	1.2 ± 0.7	1.2 ± 0.7
Microalbumin (mg/L)	212.0 ± 323.4	212.4 ± 324.2
Beta-2-microglobulin (*μ*g/L)	1771.3 ± 3269.7	1772.4 ± 3275.0
Amylase (IU/L)	298.2 ± 245.2	300.3 ± 246.5
*N*-acetyl-*β*-D-glucosaminidase (U/L)	17.4 ± 19.2	17.4 ± 19.2
Urea nitrogen (mg/dL)	620.2 ± 315.9	623.3 ± 317.8
Uric acid (mg/dL)	49.8 ± 27.5	50.0 ± 27.7
Calcium (mg/dL)	10.7 ± 9.7	10.8 ± 9.7
Magnesium (mg/dL)	6.8 ± 3.8	6.8 ± 3.8
Sodium (mEq/L)	106.9 ± 48.9	107.1 ± 49.1
Potassium (mEq/L)	45.4 ± 26.3	45.5 ± 26.4
Chlorine (mEq/L)	169.5 ± 85.4	169.7 ± 85.2
Inorganic phosphorus (mg/dL)	53.1 ± 30.3	53.4 ± 30.6

## Abbreviations

MPC: 2-methacryloyloxyethyl phosphorylcholine,
poly(MPC*-co-*BMA): poly(MPC*-co-*n-butyl methacrylate(BMA)).
